# 3-Deoxysappanchalcone Inhibited High Mobility Group Box Protein 1-Mediated Severe Inflammatory Responses

**DOI:** 10.3390/ph18050731

**Published:** 2025-05-16

**Authors:** Jinhee Lee, Gyuri Han, Jong-Sup Bae

**Affiliations:** College of Pharmacy, CMRI, Research Institute of Pharmaceutical Sciences, Kyungpook National University, Daegu 41566, Republic of Korea; aadd8563@gmail.com (J.L.); f11074@naver.com (G.H.)

**Keywords:** 3-deoxysappanchalcone, barrier integrity, LPS, HMGB1, endothelium

## Abstract

**Background/Objectives:** Phytochemicals are increasingly recognized for their therapeutic potential in treating various diseases, including vascular disorders. High mobility group box 1 (HMGB1), a key mediator of late-stage sepsis, triggers the release of proinflammatory cytokines, leading to inflammation and systemic complications. Elevated plasma levels of HMGB1 impair diagnosis and prognosis while worsening outcomes in inflammatory conditions. 3-deoxysappanchalcone (3-DSC), a compound derived from *Biancaea sappan* (L.) Tod., has demonstrated anti-influenza and anti-allergic effects, though its role in HMGB1-mediated severe vascular inflammation remains unclear. This study hypothesized that 3-DSC could modulate lipopolysaccharide-induced HMGB1 activity and its downstream inflammatory pathways in human umbilical vein endothelial cells (HUVECs). **Methods**: In vitro and in vivo permeability; cell viability, adhesion, and excavation of leukocytes; the development of cell adhesion molecules; and lastly, the production of proinflammatory substances were investigated on human endothelial cells and mouse disease models to investigate the efficacy of 3-DSC in inflammatory conditions. **Results**: Experiments revealed that 3-DSC inhibited HMGB1 translocation from HUVECs, reduced neutrophil adhesion and extravasation, suppressed HMGB1 receptor formation, and blocked nuclear factor-κB (NF-κB) activation and tumor necrosis factor-α (TNF-α) synthesis. **Conclusions**: These findings suggest that 3-DSC effectively mitigates HMGB1-driven inflammation, offering promise as a therapeutic candidate for inflammatory diseases.

## 1. Introduction

Under normal physiological conditions, high mobility group box protein 1 (HMGB1) resides within intracellular spaces, but when secreted extracellularly, it functions as a potent proinflammatory mediator [1]. Elevated cytoplasmic levels of HMGB1 serve as an indicator of cellular or tissue damage and act as a signaling molecule to alert innate immune cells of infection [1]. Beyond its role as a damage-associated molecular pattern (DAMP), HMGB1 activates membrane-bound receptors such as toll-like receptors (TLR-2 and TLR-4) and the receptor for advanced glycation end-products (RAGE) [2]. This activation triggers downstream pathways, including nuclear factor-κB (NF-κB) and extracellular signal-regulated kinases (ERK1/2), which subsequently enhance the production of cell adhesion molecules (CAMs) and proinflammatory proteins in the extracellular matrix [2].

Plant-derived bioactive compounds are gaining recognition as effective alternatives to synthetic anticoagulants due to their reduced side effects and therapeutic potential [3]. Among these, 3-deoxysappanchalcone (3-DSC), a chalcone derivative isolated from the heartwood of *Biancaea sappan* (L.) Tod., is one of several phenolic constituents alongside brazilin, protosappanin, and homoisoflavonoids, all known for their diverse biological activities [4]. Extracts from *B*. *sappan* have demonstrated a wide range of pharmacological properties, including anti-inflammatory, antioxidant, anti-influenza, anti-allergic, immunomodulatory, and hepatoprotective effects [5,6,7,8,9]. Furthermore, toxicological studies have confirmed the safety of *B*. *sappan* extracts for therapeutic use [10].

Although anti-HMGB1 antibodies have shown protective effects in improving survival during endotoxemia in mice, their application in animal models remains limited due to concerns regarding safety and efficacy [11]. These limitations arise from the overestimation of their effectiveness and the underestimation of potential toxic hazards in humans, leading to reduced therapeutic success even in experimental settings. In contrast, natural compounds derived from herbal sources, which can mitigate HMGB1-driven inflammatory responses without causing cellular toxicity, present a promising alternative for drug development. Despite the therapeutic promise of anti-HMGB1 antibodies, their clinical application is limited by safety and efficacy concerns, highlighting the need for alternative strategies [11]. 3-DSC offers a promising approach due to its anti-inflammatory and antioxidant properties and favorable safety profile. This study is significant as it demonstrates that 3-DSC effectively suppresses HMGB1 release, preserves endothelial barrier integrity, and inhibits key inflammatory pathways in both in vitro and in vivo models. By elucidating the mechanisms through which 3-DSC mitigates HMGB1-mediated vascular inflammation, this research provides a foundation for the development of novel, plant-based therapeutics targeting severe inflammatory diseases, addressing an urgent unmet medical need. Based on this rationale, we hypothesized that 3-DSC could regulate HMGB1 synthesis and activity under septic conditions both in vitro and in vivo. This study aimed to evaluate whether 3-DSC could suppress HMGB1 release induced by endotoxin and its subsequent effects on endothelial permeability, severe inflammatory responses, and underlying molecular mechanisms. The originality of this study lies in its comprehensive investigation of 3-DSC as a novel inhibitor of HMGB1-mediated severe inflammatory responses in vascular endothelial cells and in vivo models.

## 2. Results

### 2.1. 3-DSC Reduced LPS-Mediated HMGB1 Release and HMGB1-Mediated Barrier Disruptive Responses

**Figure 1:**
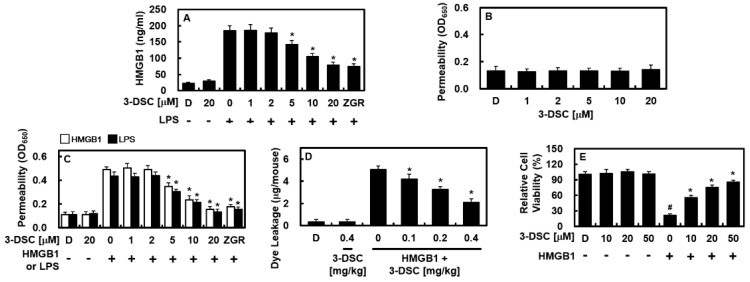
Effect of 3-DSC on the release of HMGB1 and on HMGB1-mediated permeability in vitro and in vivo. (**A**) HUVECs were exposed to LPS (100 ng/mL) for 6 h, followed by treatment with varying concentrations of 3-DSC for 16 h. HMGB1 release was quantified using ELISA. (**B**) Endothelial permeability was assessed by measuring the flux of Evans blue-bound albumin across HUVEC monolayers treated with different concentrations of 3-DSC. (**C**) HUVECs were pretreated with HMGB1 (white bars, 1 μg/mL) or LPS (black bars, 100 ng/mL) for 6 h to induce hyperpermeability, followed by treatment with 3-DSC for 16 h. (**E**) The effects of 3-DSC on HMGB1-induced vascular hyperpermeability in mice (2 μg/mouse, intravenous injection; n = 5) was evaluated by quantifying the Evans blue content in peritoneal washings (expressed as μg/mouse). DMSO (0.5%) served as the vehicle control. Statistical analysis was performed using one-way ANOVA with Tukey’s post hoc test. Results are presented as mean ± SD from three independent experiments (n = 3). * *p* < 0.05 compared to LPS-treated cells (**A**,**C**), HMGB1 alone (**B**–**D**) and # *p* < 0.05 compared to DMSO control (**E**).

Lipopolysaccharide (LPS) is widely recognized as a strong proinflammatory agent that triggers inflammatory responses, with extracellular LPS binding to HMGB1 [12]. The HMGB1-LPS complex is transported to the endolysosomal system, where it promotes HMGB1 release and severe inflammation, potentially resulting in multiple organ failure [12]. Research has demonstrated that LPS relies on HMGB1 to amplify inflammatory responses, leading to promising therapeutic strategies targeting HMGB1 in preclinical sepsis models [12]. Earlier studies identified 100 ng/mL of LPS as the optimal concentration for inducing HMGB1 secretion in endothelial cells [13], which aligns with the findings of this study (Figure 1A). In this research, Zingerone (ZGR, 20 μM) served as a positive control for anti-inflammatory effects to benchmark the activity of 3-DSC [14]. To investigate the impact of 3-DSC on LPS-induced HMGB1 release, HUVECs were exposed to LPS (100 ng/mL) for 6 h and then treated with varying concentrations of 3-DSC (1–20 μM) for 16 h. As shown in Figure 1A, 3-DSC effectively inhibited HMGB1 secretion by HUVECs (upto 58%), with significant suppression observed at concentrations exceeding 5 μM.

A permeability assay was conducted to evaluate the specific effects of 3-DSC on the barrier integrity of HUVECs activated by HMGB1. The findings revealed that 3-DSC alone did not alter endothelial barrier integrity (Figure 1B). Since HMGB1 is known to compromise endothelial barrier function [15,16], HUVECs were pretreated with 1 μg/mL of HMGB1 for 6 h and then exposed to varying concentrations of 3-DSC for 16 h. As illustrated in Figure 1C, 3-DSC effectively mitigated HMGB1-induced barrier disruption in a dose-dependent manner (upto 68%). This protective effect of 3-DSC on the endothelial barrier was further validated using LPS as a stimulus (Figure 1C, upto 69%)). To confirm these in vitro findings, the impact of 3-DSC on vascular permeability was assessed in vivo. As shown in Figure 1D, 3-DSC significantly reduced HMGB1-induced peritoneal dye leakage (upto 57%). Additionally, cell viability assays were performed to assess the potential cytotoxicity of 3-DSC on HUVECs. According to Figure 1E, concentrations of up to 50 μM of 3-DSC had no adverse effect on cell viability. Moreover, it was observed that 3-DSC could attenuate HMGB1-induced cytotoxicity (Figure 1E).

### 2.2. Antioxidant Activity of 3-DSC

**Figure 2:**
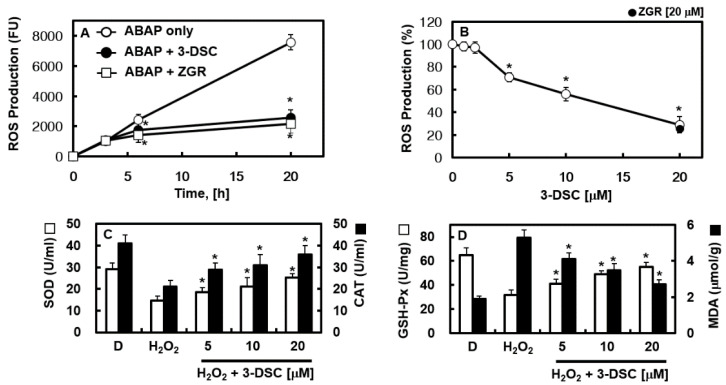
Antioxidant activity of 3-DSC. (**A**,**B**) After incubation with 3-DSC (20 μM) for 16 h, HUVECs were loaded with DCH-DA for 30 min, rinsed with PBS, and treated with 0.6 mM ABAP. ROS levels were assessed at specific time intervals. (**A**) ROS measurements were taken after treatment with 20 μM 3-DSC. (**B**) ROS production was evaluated 20 h post-ABAP addition. (**C**,**D**) HUVECs were treated with H_2_O_2_ (100 μM) for 24 h before being exposed to different concentrations of 3-DSC for an additional 16 h. ELISA was used to measure SOD, CAT, GSH-Px, and MDA levels in the cells. Statistical analysis was performed using ANOVA followed by Tukey’s post hoc test, and data are presented as the mean ± SD from three independent experiments (n = 3). * *p* < 0.05 compared to ABAP alone (**A**), untreated cells (**B**), or H_2_O_2_ alone (**C**,**D**).

The study investigated the antioxidant properties of 3-DSC to evaluate its effectiveness in penetrating cells and mitigating oxidative stress caused by ABAP-derived peroxyl radicals, as described by Wolfe et al. [17]. The experiment assessed the protective capabilities of the tested substances through mechanisms such as radical scavenging and bioactivity enhancement. Figure 2A illustrates the kinetics of DCH oxidation in HUVECs exposed to peroxyl radicals generated by ABAP, showing that radical production increased over time but was significantly regulated by 3-DSC treatment (upto 66%). Additionally, Figure 2B reveals that 3-DSC suppressed ROS-induced fluorescence elevation in a dose-dependent manner (upto 71%). To explore the impact of 3-DSC on oxidative damage in HUVEC cells, levels of SOD, CAT, GSH-Px, and MDA were measured in the culture supernatants. Oxidative stress caused by H_2_O_2_ led to a notable reduction in SOD and GSH-Px activities compared to the control group, highlighting impaired antioxidant defense in cells. However, treatment with 3-DSC at concentrations of 10 and 20 μM significantly restored the activities of SOD (upto 86%), CAT (upto 87%), and GSH-Px (upto 85%) relative to the H_2_O_2_ group. Furthermore, MDA levels were significantly elevated in the H_2_O_2_ group but were markedly reduced following 3-DSC treatment (upto 49%) (Figure 2C,D). These findings confirm that 3-DSC effectively permeates cell membranes and acts as a potent scavenger of hydroperoxyl radicals within cells.

### 2.3. 3-DSC Suppressed HMGB1-Mediated CAM Expression, Adhesion, and Migration of Neutrophils

**Figure 3:**
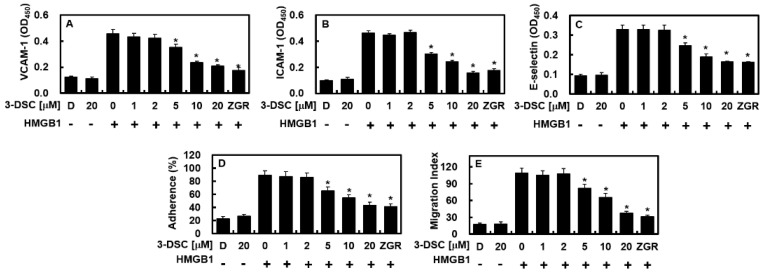
Effect of 3-DSC on HMGB1-mediated CAM expressions, cell adhesion, and TEM. The expression levels of VCAM-1 (**A**), ICAM-1 (**B**), and E-selectin (**C**) in HUVECs stimulated with HMGB1 (1 μg/mL) were evaluated following treatment with various concentrations of 3-DSC for 16 h. (**D**) HUVEC monolayers were exposed to HMGB1 (1 μg/mL) for 6 h before being treated with different concentrations of 3-DSC for 16 h, and neutrophil adhesion to the HUVECs was quantified. (**E**) Neutrophil migration across HMGB1-stimulated HUVEC monolayers was assessed after treatment with indicated concentrations of 3-DSC for 16 h. DMSO (0.5%) was used as the vehicle control. Statistical analysis was performed using one-way ANOVA followed by Tukey’s post hoc test, and results are expressed as mean ± SD from three independent experiments (n = 3). * *p* < 0.05 compared to HMGB1-treated cells.

HMGB1 plays a crucial role in inflammatory processes by upregulating the expression of adhesion molecules, including ICAM-1, VCAM-1, and E-selectin, on the surface of endothelial cells. This upregulation facilitates the transmigration of leukocytes across the endothelium in inflamed tissues [18,19]. As shown in Figure 3A–C, HMGB1 significantly increased the expression of these adhesion molecules on the cell membrane, while 3-DSC effectively inhibited this effect in a dose-dependent manner (VCAM, upto 54%; ICAM, 66%; E-selectin, 49%;). The ability of 3-DSC to modulate CAM expression suggests its involvement in disrupting the HMGB1 signaling pathway. The elevated levels of CAMs were strongly correlated with enhanced neutrophil adhesion to HMGB1-activated HUVECs and subsequent neutrophil migration [18]. However, treatment with 3-DSC reduced both neutrophil adhesion (upto 52%) and migration (upto 67%) across the activated endothelial layer in a dose-dependent manner (Figure 3D,E). These findings demonstrate that 3-DSC effectively inhibits HMGB1-driven neutrophil adhesion and transendothelial migration during inflammation.

### 2.4. 3-DSC Inhibited HMGB1-Mediated NF-κB Expression and TNF-α Production

**Figure 4:**
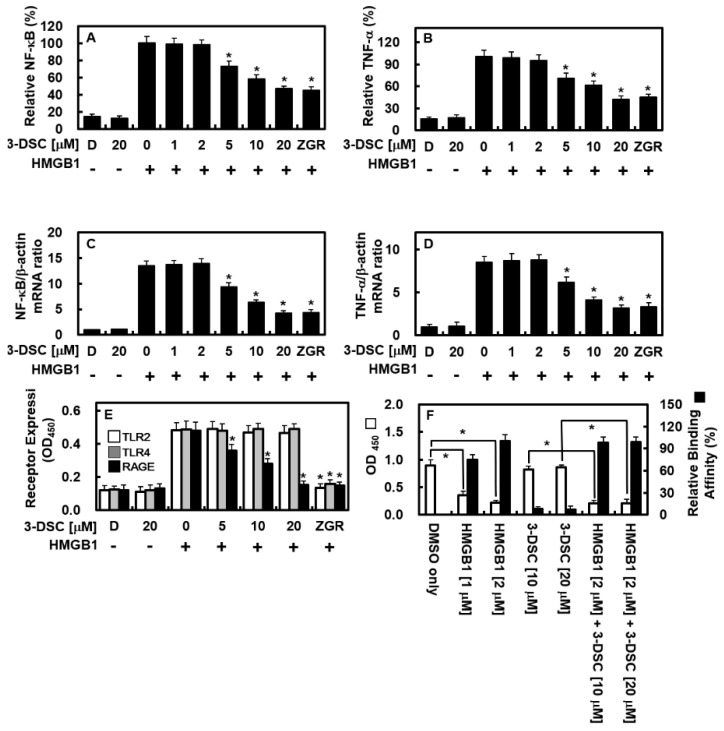
Effect of 3-DSC on HMGB1-mediated NF-κB expression, TNF-α production, and expression of pattern recognition receptors on HUVECs. (**A**–**D**) HUVEC monolayers were treated with HMGB1 (1 μg/mL) followed by exposure to various concentrations of 3-DSC for 16 h. NF-κB expression (**A**) and TNF-α production (**B**) were quantified using ELISA, while relative mRNA levels of NF-κB (**C**) and TNF-α (**D**) were analyzed via qPCR. (**E**) HUVECs were stimulated with HMGB1 (1 μg/mL for 6 h) and subsequently treated with 3-DSC for 16 h. The expression levels of TLR2 (white bars), TLR4 (gray bars), and RAGE (black bars) on the cell surface were evaluated using a cell-based ELISA. (**F**) The ability of 3-DSC to interfere with HMGB1 binding to RAGE was assessed through competitive ELISA, measuring absorbance at OD450 (white bars). Binding affinity of HMGB1 (2 μM) was set as the baseline at 100%, and relative binding affinities were calculated accordingly (black bars). DMSO (0.5%) served as the vehicle control. Statistical analysis was performed using one-way ANOVA followed by Tukey’s post hoc test, with data presented as mean ± SD from three independent experiments (n = 3). * *p* < 0.05 compared to HMGB1-treated cells (**A**–**E**).

NF-κB plays a central role in initiating the proinflammatory cascade, while both NF-κB and TNF-α are key contributors to inflammatory signaling in endothelial cells [20,21]. Moreover, HMGB1 is known to promote the production of TNF-α and the activation of NF-κB [22,23]. To explore whether 3-DSC could regulate these inflammatory pathways, we examined its effects on HMGB1-induced NF-κB activation and TNF-α production in HUVECs. HMGB1 significantly increased the expression of NF-κB and TNF-α but treatment with 3-DSC effectively suppressed the expression of NF-κB (Figure 4A, upto 53%) and TNF-α (Figure 4B, upto 58%). Additionally, qPCR analysis confirmed that 3-DSC reduced mRNA levels of NF-κB (Figure 4C, upto 69%) and TNF-α (Figure 4D, upto 64%). These findings indicate that 3-DSC targets two essential signaling molecules involved in mediating proinflammatory responses in endothelial cells.

### 2.5. 3-DSC Downregulated RAGE Expression

To investigate the impact of HMGB1 on receptor expression and the regulatory effects of 3-DSC, we examined the expression levels of TLR2, TLR4, and RAGE in endothelial cells. As shown in Figure 4E, HMGB1 significantly increased the expression of all three receptors in HUVECs, while 3-DSC selectively reduced RAGE expression (upto 69%) without affecting TLR2 or TLR4. To determine whether 3-DSC interferes with the HMGB1–RAGE interaction, a modified competitive ELISA was performed. Figure 4F demonstrates that 3-DSC neither bound directly to RAGE nor disrupted the interaction between HMGB1 and RAGE. These findings indicate that 3-DSC specifically inhibits HMGB1-induced RAGE expression but does not directly interfere with the binding between HMGB1 and RAGE.

## 3. Discussion

This study proposes that 3-DSC has the potential to regulate HMGB1 production under inflammatory conditions both in vitro and in vivo, while also mitigating HMGB1-driven proinflammatory responses in endothelial cells. This hypothesis was supported by findings showing that 3-DSC effectively inhibited HMGB1 release from HUVECs and reduced HMGB1-induced neutrophil adhesion and migration to activated endothelial cells. Furthermore, 3-DSC decreased the expression of HMGB1 receptors and suppressed HMGB1-mediated inflammatory signaling by attenuating NF-κB activity and TNF-α expression triggered by HMGB1.

The hyperacetylation of HMGB1 is essential for its ability to bind DNA or cellular plasma and drives its cytoplasmic translocation by inhibiting nuclear localization [24]. Conversely, SIRT1, a deacetylase, targets HMGB1 for deacetylation, making it a novel substrate for SIRT1 activity [25]. Previous studies have demonstrated that herbal compounds such as cornuside, hederacolchiside-E, rare ginsenosides, JH-4 (a synthetic decursin derivative), and aloin enhance SIRT1 expression and facilitate HMGB1 deacetylation [26,27,28,29,30]. Based on these findings, it is plausible that 3-DSC may similarly promote HMGB1 deacetylation and activate SIRT1 expression, warranting further investigation to confirm this hypothesis.

Traditional Chinese herbal medicines have long been utilized for their therapeutic benefits, characterized by clinical efficacy, extensive knowledge, high safety, and minimal toxicity. Natural compounds derived from plants are widely acknowledged as effective agents for managing inflammatory conditions [31,32]. These plant-based chemicals provide a foundation for developing novel pharmacological treatments due to their consistent molecular structures [31]. In this study, 3-DSC was found to inhibit LPS-induced HMGB1 secretion in HUVECs and suppress HMGB1-mediated proinflammatory responses. The anti-inflammatory activity of 3-DSC was most pronounced at concentrations above 5 μM, as evidenced by its ability to reduce HMGB1 release. Since vascular barrier disruption is a hallmark of many inflammatory diseases [33], the study highlights the role of 3-DSC in preserving endothelial barrier integrity by mitigating HMGB1-induced permeability changes. This protective effect underscores the importance of 3-DSC in maintaining vascular stability during inflammation.

The elevated expression of adhesion molecules such as ICAM-1, VCAM-1, and E-selectin on endothelial cell surfaces is a hallmark of proinflammatory activity [34]. Notably, inflammation is characterized by two key processes: the adhesion of leukocytes to the damaged endothelial layer and their subsequent migration across it [35,36]. Targeting these processes by reducing adhesion molecule expression and leukocyte migration represents a promising strategy for combating inflammation. By inhibiting HMGB1-induced CAM expression, 3-DSC effectively disrupted neutrophil adhesion to activated endothelial cells and prevented their transendothelial migration, highlighting its potential as a potent anti-inflammatory agent.

The in vitro findings demonstrated that 3-DSC effectively reduced HMGB1 levels in HUVECs treated with HMGB1, which is closely linked to the NF-κB signaling pathway and TNF-α production. Additionally, 3-DSC inhibited the NF-κB pathway, thereby suppressing HMGB1 expression and its associated proinflammatory effects. Notably, 3-DSC also decreased the expression of RAGE, the primary HMGB1 receptor displayed on the surface of HUVECs [36,37]. Interestingly, while 3-DSC significantly inhibited HMGB1-in6duced RAGE expression, it did not directly bind to RAGE or interfere with the HMGB1–RAGE interaction.

Elevated plasma levels of HMGB1 have been detected in patients with acute inflammatory conditions and in animal models treated with endotoxins [38]. This increase in HMGB1 correlates with its role as a poor prognostic marker for survival in severe inflammatory diseases like sepsis [38]. As a late mediator of inflammation, HMGB1 is released during the advanced stages of endotoxemia in rodents [11]. Experimental studies have shown that administering HMGB1 to animals results in severe intestinal tissue damage and poses a significant threat to [11,15,16]. Conversely, the use of anti-HMGB1 antibodies has been effective in reducing acute inflammation and improving survival rates in animal models rodents [11]. These findings highlight HMGB1’s critical role in driving late-stage inflammatory responses, leading to tissue injury, microvascular thrombosis, and organ failure. Furthermore, evidence from this study suggests that human vascular endothelial cells are key sources of extracellular HMGB1, which is released in response to pathological endotoxins and proinflammatory stimuli. Importantly, 3-DSC has shown promise in suppressing HMGB1 release, offering potential therapeutic benefits for managing various inflammatory disorders.

The findings of this study revealed that 3-DSC effectively inhibited LPS-induced HMGB1 secretion and alleviated HMGB1-induced endothelial barrier damage by preserving barrier integrity and reducing CAM expression, thereby limiting leukocyte adhesion and migration. Additionally, 3-DSC demonstrated antioxidant properties in HUVECs by suppressing HMGB1-associated NF-κB activation and TNF-α production, both of which are key inflammatory markers. Moreover, 3-DSC significantly downregulated RAGE expression induced by HMGB1. These results highlight the potential of 3-DSC as a promising therapeutic agent for managing vascular inflammation. Future in vivo studies using mouse models with HMGB1-induced inflammation are needed to validate the clinical efficacy of 3-DSC in treating vascular inflammatory diseases.

## 4. Materials and Methods

### 4.1. Reagents

3-DSC (purity > 95%) was obtained from MuseChem (Fairfield, NJ, USA). Lipopolysaccharide derived from bacteria (LPS, serotype: 0111:B4, L5293), zingerone which is used as positive control [14] (ZGR, purity > 96%), Evans blue, crystal violet, 2-mercaptoethanol, and penicillin G and streptomycin were commercially obtained from Sigma (St. Louis, MO, USA). Human recombinant HMGB1 was provided by Abnova (Taipei City, Taiwan), and Vybrant DiD and FBS (fetal bovine serum) were provided by Invitrogen (Carlsbad, CA, USA).

### 4.2. Cell Culture

Primary human umbilical vein endothelial cells (HUVECs) were obtained from Cambrex Bio Science (Charles City, IA, USA) and cultured following previously established protocols [14]. Additionally, human neutrophils were isolated from 15 mL of peripheral blood collected from healthy volunteers (n = 5) and processed as described in earlier studies [14]. The study protocol was approved by the Institutional Review Board of Kyungpook National University Hospital (KNUH 2019-01-010) in Daegu, Republic of Korea.

### 4.3. Animals and Husbandry

Male C57BL/6 mice, aged 6–7 weeks and weighing approximately 27 g, were purchased from Orient Bio Co. (Sungnam, Republic of Korea) and acclimated for 12 days prior to experimentation. The mice were housed in groups of five per cage under controlled environmental conditions, including a temperature range of 20–25 °C, humidity levels of 40–45%, and a 12 h light/dark cycle. They were provided with standard rodent chow and water ad libitum during the acclimatization period. All animal care and experimental procedures adhered to the “Guidelines for the Care and Use of Laboratory Animals” and were approved by the Institutional Review Board of Kyungpook National University (IRB No. KNU 2024-13).

### 4.4. Cell Viability Assay

To evaluate cell viability, an MTT assay [3-(4,5-dimethylthiazol-2-yl)-2,5-diphenyltetrazolium bromide] was performed following previously described methods [14]. HUVECs were seeded into 96-well plates at a density of 5 × 10^3^ cells per well and incubated for 24 h. Subsequently, the cells were exposed to HMGB1 (1 µg/mL) for 6 h.

### 4.5. Competitive Enzyme-Linked Immunosorbent Assay for HMGB1

A competitive enzyme-linked immunosorbent assay (ELISA) was performed to measure HMGB1 levels in the culture medium of HUVECs, following previously established methods [26]. HUVECs were activated with lipopolysaccharide (LPS, 100 ng/mL) for 6 h, after which they were washed three times with phosphate-buffered saline (PBS) and treated with 3-DSC for an additional 16 h. The culture supernatants were then collected to determine HMGB1 concentrations. A modified competitive ELISA protocol was used to quantify HMGB1 expression as described in earlier studies [26].

### 4.6. Permeability Assay In Vivo

Male mice were anesthetized using isoflurane (Forane 2%, JW Pharmaceutical, Seoul, Republic of Korea) in an oxygen-supplied gas anesthesia system designed for experimental mice (RC2, Vetequip, Pleasanton, CA, USA). Initially, the mice were placed in a respiratory chamber, followed by the use of a face-covering mask. The anesthetized mice maintained normal breathing throughout the procedure and were intravenously injected with HMGB1 (2 μg/mouse) for 6 h before receiving 3-DSC at doses of 0.1, 0.2, or 0.4 mg/kg. Given that the average circulating blood volume in mice is 72 mL/kg [14], and their average weight and blood volume were approximately 27 g and 2 mL, respectively, the administered doses corresponded to maximum blood concentrations of 5, 10, or 20 μM. After 16 h, each mouse was injected intravenously with a mixture of Evans blue dye (1%) and normal saline. Thirty minutes later, the mice were euthanized by cervical dislocation, and peritoneal cavity leakage was collected by flushing with 5 mL of normal saline. The collected fluid was centrifuged at 200× *g* for 10 min, and the absorbance of the supernatant was measured at 650 nm. Vascular permeability was quantified as the microgram amount of Evans blue dye per peritoneal tissue sample and analyzed using a standard curve, as previously described [26].

### 4.7. Permeability Assay In Vitro

The permeability of HUVEC monolayers was assessed using varying concentrations of 3-DSC to counteract HMGB1-induced hyperpermeability in vitro. Changes in the passage of Evans blue-albumin complexes across the cell layers were measured using a modified two-chamber Transwell system, as previously described [14]. HUVECs (5 × 10^4^ cells/well) were seeded into the upper compartment of Transwell inserts (3 μm pore size, Corning, Lowell, MA, USA) and cultured for 72 h. Confluent HUVEC monolayers were exposed to HMGB1 (1 μg/mL) or LPS (100 ng/mL) for 6 h to induce permeability changes. After washing the cells three times with PBS, they were incubated with media containing 3-DSC for an additional 16 h.

### 4.8. ELISA for HMGB1, Nuclear Factor-κB (NF-κB), and Tumor Necrosis Factor-α (TNF-α)

To detect the level of NF-κB, we used an ELISA kit (Cell Signaling Technology, Danvers, MA, USA) for nuclear extracts and TNF-α (R&D Systems, Minneapolis, MN, USA) for supernatants following the companies’ instructions.

### 4.9. Antioxidant Effects of 3-DSC on HUVECs

Reactive oxygen species (ROS) generation in cells was quantified using the non-fluorescent compound 2′,7′-dichlorofluorescein diacetate (DCH-DA). Inside the cells, DCH-DA was enzymatically hydrolyzed by esterases and subsequently oxidized to form 2′,7′-dichlorofluorescein (DCH), which fluoresces upon reacting with ROS. Peroxyl radicals were produced by thermal decomposition of 2,2′-azobis(2-amidinopropane) (ABAP), which degraded at a rate of approximately 1.36 × 10^−6^ s^−1^ at 37 °C, releasing up to 1 × 10^12^ radicals/mL/s [39,40]. HUVECs were cultured in 24-well plates at a density of 3 × 10^6^ cells per well and treated with 3-DSC for 16 h. The cells were then incubated with DCH-DA for 30 min, washed twice with PBS, and exposed to ABAP (0.6 mM). Fluorescence measurements, indicative of ROS levels, were performed using a spectrometer with excitation at 485 nm and emission at 520 nm. To evaluate oxidative stress markers such as superoxide dismutase (SOD), catalase (CAT), glutathione peroxidase (GSH-Px), and malondialdehyde (MDA), commercial assay kits from LSBio (Seattle, WA, USA) were employed following the manufacturer’s protocols. For these assays, HUVECs were plated in either 96-well plates at a density of 1 × 10^5^ cells/mL for MDA analysis or in 6-well plates at a density of 2 × 10^5^ cells/mL for SOD, CAT, and GSH-Px measurements. After a 24 h incubation period, the cells were treated with varying concentrations of 3-DSC (5, 10, and 20 μM) for 16 h and subsequently exposed to H_2_O_2_ (100 μM) for another 24 h to induce oxidative damage. The supernatant was collected to measure MDA levels using ELISA. For SOD, CAT, and GSH-Px assays, the cells were detached with 0.25% trypsin and subjected to ultrasonic lysis to extract the cytosolic fraction. Enzymatic activities of SOD, CAT, and GSH-Px in the cytosol were then determined via ELISA.

### 4.10. Cell–Cell Adhesion Assay

Neutrophils isolated from human blood (1.5 × 10^6^ cells/mL, 200 μL per well) were labeled using Vybrant DiD dye and introduced to HUVECs following established protocols [14]. The HUVEC monolayers were treated with HMGB1 (1 μg/mL) or LPS (100 ng/mL) for 6 h to induce stimulation. After washing the HUVECs three times with PBS, they were exposed to 3-DSC for 16 h. Neutrophils that firmly adhered to the HUVECs remained attached, while loosely bound neutrophils were removed during washing. The percentage of adherent neutrophils was calculated using the following formula: % adherence = (signal from adherent neutrophils/signal from total neutrophils) × 100.

### 4.11. Transendothelial Migration (TEM) Assay

Transwell inserts (6.5 mm diameter, 8 μm pore size; Corning, Lowell, MA, USA) were utilized to evaluate cell translocation. HUVECs (6 × 10^4^ cells) were cultured for three days to form confluent endothelial monolayers. The monolayers were stimulated with HMGB1 (1 μg/mL) or LPS (100 ng/mL) for 6 h, followed by three PBS washes before being treated with 3-DSC for 16 h. Neutrophils were then added to the upper chamber of the Transwell system and allowed to interact with the HUVECs for 2 h. After incubation, cells in the upper chamber were removed, and any remaining cells on the filter surface were carefully wiped off using a swab. Immune cells that migrated through the filter were fixed with 8% glutaraldehyde and stained with 0.25% crystal violet prepared in 20% methanol (*w*/*v*). Each experiment was performed in duplicate under identical conditions, and cell migration was quantified by imaging and counting cells in nine randomly selected high-power microscopic fields per well. The results reflect the extent of cell migration.

### 4.12. Expression of Cell Adhesion Molecules (CAMs) and HMGB1 Receptors

HUVEC monolayers were treated with HMGB1 (1 μg/mL) for 6 h, washed three times with PBS, and subsequently incubated with varying concentrations of 3-DSC (1–20 μM) for 16 h. The levels of intercellular adhesion molecule-1 (ICAM-1), vascular cell adhesion molecule-1 (VCAM-1), E-selectin, TLR2, TLR4, and RAGE expressed on HUVECs were measured using ELISA kits, following the instructions provided by the manufacturer (LSBio).

### 4.13. Quantitative Real-Time PCR (qPCR)

RNA was isolated using TRI Reagent (Invitrogen, Carlsbad, CA, USA), and the extracted RNA was subjected to reverse transcription in a PX2 Thermal Cycler (Thermo Scientific, Waltham, MA, USA). The reaction was carried out in a 20 µL volume using 0.5 mg/µL oligo(dT)-adapter primers (Invitrogen) and M-MLV reverse transcriptase (Invitrogen). Expression levels of NF-κB and TNF-α were normalized to β-actin as the reference gene. For PCR analysis, custom-designed primers were used as follows: NF-κB forward, 5′-GAA TGG CTC GTC TGT AGT G-3′; NF-κB reverse, 5′-TGG TAT CTG TGC TCC TCT C-3′; TNF-α forward, 5′-CCT GGT ATG AGC CCA TCT ATC-3′; TNF-α reverse, 5′-AGG TTG AGG GTG TCT GAA G-3′; β-actin forward, 5′-CAA GAT CAT TGC TCC TCC TG-3′; β-actin reverse, 5′-ATC CAC ATC TGC TGG AAG G-3′. The thermal cycling conditions for PCR included an initial denaturation at 94 °C for 7 min, followed by 40 cycles of denaturation at 94 °C for 15 s and annealing/extension at 60 °C for 45 s.

### 4.14. Statistical Analysis

Data were presented as the mean ± standard deviation (SD) from at least three independent experiments. Statistical analysis was performed using SPSS software (version 16.0, SPSS Inc., Chicago, IL, USA). Differences between groups were evaluated through one-way analysis of variance (ANOVA) followed by Tukey’s post hoc test. A *p*-value of less than 0.05 was considered statistically significant.

## Data Availability

The data that support the findings of this study are available on request from the corresponding author. The data are not publicly available due to privacy or ethical restrictions.
